# 936. Single Southeastern State Comparison of Combined Mortality Rates Among ABA Verified to Non-verified Burn Centers

**DOI:** 10.1093/jbcr/irag033.503

**Published:** 2026-04-06

**Authors:** Briana Sylvester, Jeffrey E Carter, M Victoria P Miles, Jonathan E Schoen, Paige B Hargrove, Dave J Barrios, III

**Affiliations:** University Medical Center Burn Center, Louisiana State University Health Sciences Center New Orleans, Louisiana; University Medical Center Burn Center, Louisiana State University Health Sciences Center New Orleans, Louisiana; University Medical Center Burn Center, Louisiana State University Health Sciences Center, Department of Surgery, New Orleans, New Orleans, LA; UCI Health Regional Burn Center, New Orleans, LA; Louisiana Emergency Response Network, Baton Rouge, LA; Our Lady of Lourdes Regional Medical Center, Lafayette, LA

## Abstract

**Introduction:**

ABA verification is an indicator of high-quality patient care to burn patients from the time of injury through rehabilitation. Maintaining ABA verification incorporates principles of quality assurance and continuous improvement. Despite extensive efforts, the impact and value of verification lacks sufficient evidence in clinical outcomes. Our goal was to assess the impact of ABA verification on burn mortality across verified and non-verified burn centers.

**Methods:**

Burn admission and mortality data from 2019 through 2023 were queried from our state's Department of Health. Mortality rates at ABA-verified burn centers were analyzed against non-verified burn centers. An independent samples t-test was conducted to assess whether there was a statistically significant difference in mean mortality rates between verified and non-verified centers.

**Results:**

Over the five-year period in this state, ABA-verified centers reported a total of 398 mortality events in 5745 admissions, yielding a combined mortality rate of 6.92%. Non-verified centers experienced 124 mortality events in 1308 admissions, with a mortality rate of 9.48%. The difference in mortality rates between the verified and non-verified regions was statistically significant (*p*=.0022, 95% CI: [-0.04186, -0.00919]).

**Conclusions:**

These findings suggest that in this state mortality rates are significantly lower at ABA-verified burn centers when compared to non-verified centers. A key limitation of this analysis is the lack of data on concomitant burn and traumatic injuries, which are associated with higher mortality and may have influenced regional difference. Additionally, study populations were not matched for other parameters such as inhalation injury, burn size, or age. Future research may identify center specific practices that contribute to improved survival as well as investigate barriers to achieving ABA verification in underrepresented regions.

**Applicability of Research to Practice:**

This study explores the value offered by the verification process and most importantly, how it relates to patients.

**Funding for the study:**

N/A.

